# Cursory Notes on the Morbid Eye

**Published:** 1841-01

**Authors:** 


Art. IV.—*Cursory Notes on the Morbid Eye.* By Robert Hull, m.d., Physician to the Norfolk and Norwich Hospital.—*London*, 1840. 8vo, pp. 249.

We are told in the dedication to this "tractate" that it "contains some digressions from the main object into the region of professional morals." And truly it does contain digressions. In the dedication the good old times of the profession are indiscriminately lauded, and the present times abused; and a great part of the preface we find occupied in decrying general medical reform, but praising the College of Physicians and the late alterations in its bye-laws.

The practical point which has struck us most in the perusal of Dr. Hull's book is his extensive experience in the use of turpentine in different inflammations of the eye. He has often found turpentine useful in iritis in alternation with mercury, when neither alone proved efficient. He has found children bear the medicine better than adults, and the least disagreeable mode of taking it to be "floating on water, or weak hollands and water, or diluted gin." In traumatic ophthalmiæ, in which the iris is the most evident sufferer, Dr. Hull has "found the turpentine maintain the good character which it had earned in spontaneous and simple iritis; in rheumatic iritis; in strumous; in syphiloid." In keratitis, our author has also found turpentine of great value. He relates several cases, and then remarks, "The impression on the mind of the witnesses of the terebinthinate methodus medendi is, that it tells quicker than the mercurial or the ordinary: that this is eminently visible in the wretched scrofulous child."

In Dr. von Ammon's monography on iritis, which we have already noticed, it is well remarked, "Autocratia naturæ in iritidis decursu debilis esse solet, sæpissime nulla; artis est hunc morbum cito et tuto sanare." In a similarly just strain, Dr. Hull says that iritis is essentially formidable and should never be left in quiet, but detected and attacked as soon as possible. Dr. Hull, however, perfectly accords with Dr. Holland, that, generally, we make "no due allowance for the tendency which all parts have to assume a healthy action if left in quiet." On this Dr. Hull dwells more fully at p. 168, where he says (and the saying well deserves to be laid to heart by all young practitioners),

> "As to active interference in disorders generally by professional men, the medicine expectante appears to be as rashly neglected here as it is said to be fatally indulged across the British channel. If the French physicians do, indeed, carry their expectations too far, still it may be, of two evils, much the least; and surely they deserve credit for the adoption of a mode of practice so different from the native precipitation of the Gauls. The vis medicatrix is virtually assumed to be a poetical figment; and when once the pharmacopolist has made an entry on the premises, he fancies that his sole duty and glory consist in working without her." (p. 168.)

Notwithstanding the oddities and affectation which prevails throughout this work, there are in it many glimpses of practical shrewdness and much pertinency of remark. Besides this, the author displays considerable familiarity with Greek and Latin; but it is to be regretted that he should have so frequently prostituted this to the damage of the English language.

